# Do Specialized Cells Play a Major Role in Organic Xenobiotic Detoxification in Higher Plants?

**DOI:** 10.3389/fpls.2020.01037

**Published:** 2020-07-09

**Authors:** Armand Cavé-Radet, Mokded Rabhi, Francis Gouttefangeas, Abdelhak El Amrani

**Affiliations:** ^1^ Université de Rennes 1, CNRS/OSUR-UMR 6553, Ecosystèmes-Biodiversité-Evolution, Rennes, France; ^2^ Department of Plant Production and Protection, College of Agriculture and Veterinary Medicine, Qassim University, Qassim, Saudi Arabia; ^3^ Laboratory of Extremophile Plants, Centre of Biotechnology of Borj Cedria, Hammam-Lif, Tunisia; ^4^ Université de Rennes 1, ScanMAT - Synthèse, Caractérisation et ANalyse de la MATière, Rennes, France

**Keywords:** abiotic stress, phenanthrene, pavement cells, trichomes, salt glands, endopolyploidy

## Abstract

In the present work, we used a double cell screening approach based on phenanthrene (phe) epifluorescence histochemical localization and oxygen radical detection to generate new data about how some specialized cells are involved in tolerance to organic xenobiotics. Thereby, we bring new insights about phe [a common Polycyclic Aromatic Hydrocarbon (PAH)] cell specific detoxification, in two contrasting plant lineages thriving in different ecosystems. Our data suggest that in higher plants, detoxification may occur in specialized cells such as trichomes and pavement cells in *Arabidopsis*, and in the basal cells of salt glands in *Spartina* species. Such features were supported by a survey from the literature, and complementary data correlating the size of basal salt gland cells and tolerance abilities to PAHs previously reported between *Spartina* species. Furthermore, we conducted functional validation in two independent *Arabidopsis* trichomeless glabrous T-DNA mutant lines (GLABRA1 mutants). These mutants showed a sensitive phenotype under phe-induced stress in comparison with their background ecotypes without the mutation, indicating that trichomes are key structures involved in the detoxification of organic xenobiotics. Interestingly, trichomes and pavement cells are known to endoreduplicate, and we discussed the putative advantages given by endopolyploidy in xenobiotic detoxification abilities. The same feature concerning basal salt gland cells in *Spartina* has been raised. This similarity with detoxification in the endopolyploid liver cells of the animal system is included.

## Introduction

Worldwide environmental accumulation of organic xenobiotics is a major concern for natural ecosystems and public health. This prompted environmental scientists to explore how organisms and especially plants may reduce xenobiotics in polluted environments. As sessile organisms, it is reasonable to hypothesize that plants have evolved specific detoxifying strategies during their evolutionary history to cope with environmental toxic compounds. In plants, xenobiotic root uptake was described to occur either passively (*e.g.* by simple diffusion through a concentration gradient or aquaglyceroporins) and by slow active absorption through xenobiotic/H^+^ symporters (Collins et al., 2011; Zhan et al., 2012; Dumas et al., 2016). Transport into aerial parts for xenobiotic compartmentalization is conducted by vascular tissues such as the xylem.

Currently, characterizing defense mechanisms is helping to understand how plants respond to xenobiotics (*e.g.* high metabolic activity and the release of large amounts of free oxygen radicals, see Shiri et al., 2015; Dumas et al., 2016; Cavé-Radet et al., 2019). Indeed, the studies that addressed this topic looked at the xenome, defined as “the biosystem responsible for the detection, transport, and detoxification of xenobiotics” (Edwards et al., 2005). Based on animal detoxification mechanisms of organic xenobiotics, related metabolic pathways were explored in plants, and similar responses were described (including antioxidant responses to cope with oxidative stress). Although organic xenobiotic metabolization and transformation by plants remain incompletely characterized, a model has been proposed (Edwards et al., 2005; Edwards et al., 2011; El Amrani et al., 2015; Sun et al., 2019) with three major steps: (1) signaling, (2) transport and transformation, and (3) compartmentalization. This model involves a myriad of enzymes from the xenome, among alpha-beta hydrolases (*e.g.* peroxidases and esterases) and cytochromes P450 (CYPs) for xenobiotic hydroxylation acting on their solubility, and glutathione-S-transferases (GSTs), glycosyltransferases (GTs), and malonyltransferases (MTs) for xenobiotic conjugation with endogenous glutathione, glycosyl or malonyl groups, respectively. Transformed xenobiotics can then be either transported and stored in the vacuole by ATP-binding cassette (ABC) transporters, sequestrated by cell wall polymers, or excreted out of the cell (Kvesitadze et al., 2009; Taguchi et al., 2010; Edwards et al., 2011). Genome wide transcriptional investigations in *Arabidopsis* have shown massive and rapid transcriptional changes in response to xenobiotics (Weisman et al., 2010; Dumas et al., 2016), especially in xenome genes. Beside xenobiotics cytotoxicity, oxidative stress damages related with reactive oxygen species (ROS) accumulation under abiotic stress are most likely limiting plant tolerance abilities (Liu et al., 2009).

Today, most of the available data about organic xenobiotic detoxification in higher plants point out chemical transformation followed by compartmentalization of xenobiotic conjugates (Edwards et al., 2005; Edwards et al., 2011; El Amrani et al., 2015). In some cases, xenobiotics are incorporated into macromolecules such as lignin or proteins. Hence, it has been reported that more than 70% of absorbed organic pollutants are accumulated in the form of conjugates (Öztürk et al., 2016). In this perspective, the idea through specialized cells may represent key components involved in the detoxification of organic xenobiotics has been proposed during the past decade, such as for trichomes in response to Polycyclic Aromatic Hydrocarbons (PAHs) (Alkio et al., 2005; Shiri et al., 2015; Alves et al., 2017). Trichomes (from Ancient Greek *thríx*, “hair”) are shoot hairs derived from the epidermal cell layer. They were described in most plants and can be either uni- or multicellular structures. Some trichomes are secretory glandular while others are non-glandular (Hülskamp, 2004). These structures were shown to be central to the detoxification of inorganic compounds such as cadmium or sulfur through compartmentalization and phytoexcretion (Wienkoop et al., 2004; Kolb et al., 2010). However, empirical data about the role of trichomes in the detoxification of organic xenobiotics such as PAHs are still fragmentary.

Among PAHs, phenanthrene (phe) represents a model molecule commonly used to investigate the physiological impact of organic xenobiotics in plants. Its concentration in natural ecosystems is highly variable and mainly influenced by anthropogenic activity, and in some polluted areas phe can be found at a saturation concentration. In this study, we investigated both cellular localizations of phe and ROS produced under oxidative stress in two contrasting plant lineages, using the diploid plant model *Arabidopsis* and species from the *Spartina* Schreb. clade (Poaceae). Based on fluorescence microscopy and investigation of cell specific induced stress, we found that trichomes and pavement cells are putative specialized cells involved in organic xenobiotic detoxification. The analysis of phe-treated leaves of three polyploid *Spartina* species revealed that the basal salt gland cells may also represent key structures in this perspective. We expanded our observations with complementary data linking salt gland cell sizes and tolerance abilities to PAHs previously reported between *Spartina* species. In addition, we performed functional validation to confirm the impact of trichomes in organic xenobiotic detoxification mechanisms, using *Arabidopsis* T-DNA glabrous mutant lines (trichomeless) characterized by GLABRA1 (AT3G27920) insertions.

To date, depicting how cell-specific lineages impact tolerance to organic xenobiotic stresses in higher plants has received little attention to the best of our knowledge. Here, we bring new insights linking specialized cells and their putative role in tolerance to organic xenobiotics, and we reviewed the literature around this topic. Finally, we pointed out that trichomes and pavement cells are known to undergo endoreduplication (Melaragno et al., 1993; Schnittger and Hülskamp, 2002; Katagiri et al., 2016), and we discussed the putative advantages given by endopolyploidy in specialized cells involved in xenobiotic detoxification.

## Results and Discussion

### Phe Accumulation and Oxidative Stress Markers Were Co-Localized in Specific Cells in *Arabidopsis* and *Spartina*

In plants, conjugation and compartmentalization appears as major steps in the detoxification process of xenobiotics. Once transported into the cells, xenobiotic compounds are mainly metabolized by xenome enzymes, reducing their toxicity in the form of conjugates before compartmentalization or excretion. In the present work, we performed both cellular localization of phe and oxidative stress markers in *Arabidopsis* and *Spartina* to assess the putative role of specialized cells in xenobiotic detoxification strategies.

**Figure 1:**
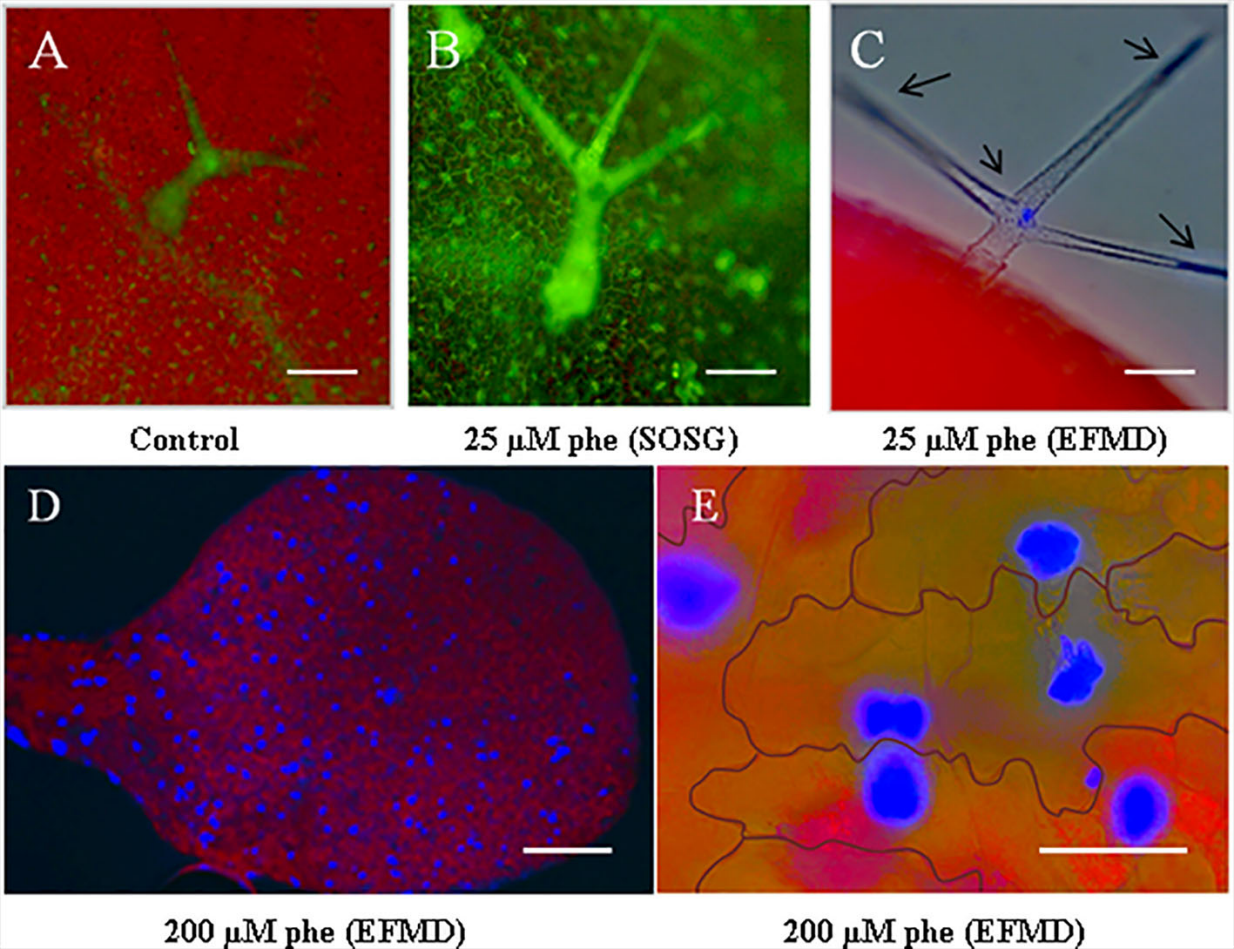
Phe visualization in trichomes and pavement cells using epifluorescence microscopy and singlet oxygen detection with Singlet Oxygen Sensor Green (SOSG) fluorescent probe. *Arabidopsis thaliana* plantlets were grown on a solid growth medium (0.5 MS) under 0 (control) and 25 µm phe. After 21 days, leaves were filtered with SOSG to detect singlet oxygen radicals as oxidative stress markers. As compared to the control **(A)**, treated leaves **(B)** revealed a pronounced generation of singlet oxygen by specific green fluorescence in trichomes. Epifluorescence microscopy detection (EFMD; **(C)** co-localized free phe sequestration in these structures, detected as blue spots (the red color corresponds to chlorophyll fluorescence). Plantlets grown on high phe content (200 µm) showed spots of phe accumulation in leaves **(D)**, localized in the pavement cells **(E)**. Scale bars = 100 µm **(A–C)**, 250 µm **(D)** and 50 µm **(E)**.

*Arabidopsis* plantlets were grown under control and moderate phe (25 µm) conditions, and leaves were filtered under vacuum with Singlet Oxygen Sensor Green (SOSG). Compared to the control (**Figure 1A**), trichomes in treated plants were found to be highly reactive to phe (**Figure 1B**), as they presented pronounced fluorescent patches, indicating a severe oxidative stress response through singlet oxygen production. Moreover, phe detected by epifluorescence microscopy pointed out a significant role of these structures in xenobiotic sequestration and detoxification (**Figure 1C**). We observed that the intensity of the phe-induced fluorescence was amplified as the concentration was increased. Indeed, the visualization of the leaf surface of plantlets grown in the presence of 200 µm phe revealed additional high fluorescent spots (**Figure 1D**). Close cellular screening showed that these spots were found to be localized specifically in the pavement cells (**Figure 1E**) from the epidermal layer. Here, we noticed that cells involved in phe metabolization/storage depend on phe concentration. In fact, in *Arabidopsis* phe was only detected in trichomes under moderate-induced stress, whereas under sublethal concentration (200 µm phe, Dumas et al., 2016), it was detected in both trichomes and pavement cells, indicating different detoxification strategies depending on the physicochemical conditions. When exceeding these concentrations, *Arabidopsis* show morphological and cellular symptoms of xenobiotic stress such as chlorosis, necrotic lesions, perturbations in signaling and metabolic pathways that regulate ROS and responses related to pathogen defense and cell death (Weisman et al., 2010; Dumas et al., 2016).

**Figure 2:**
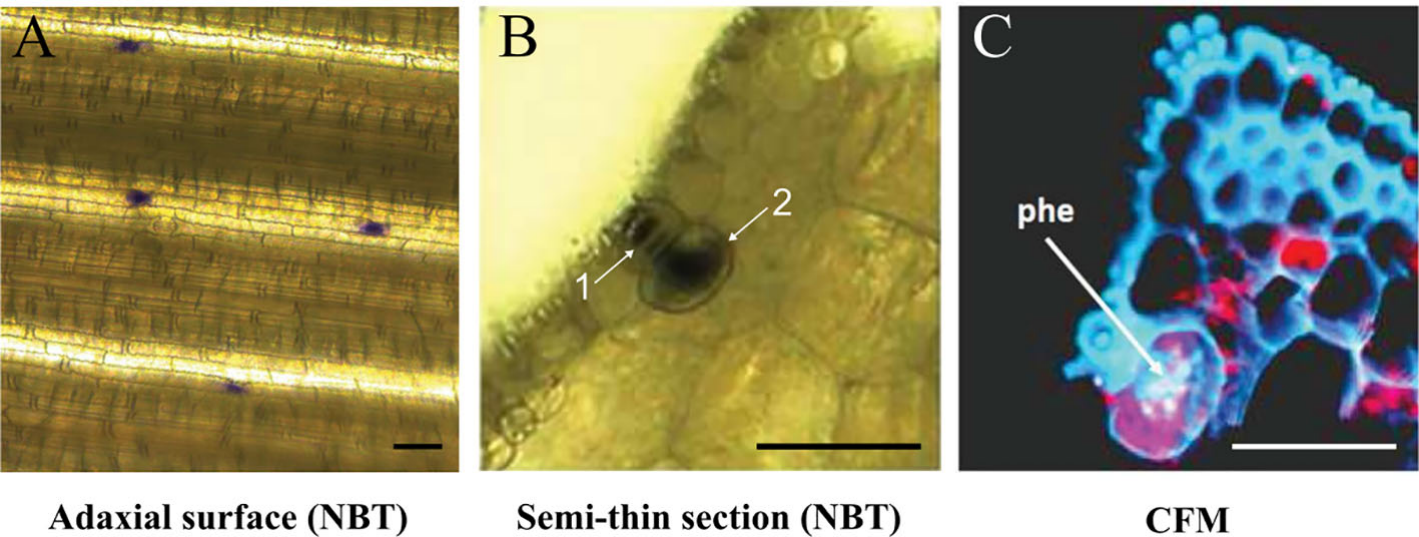
Superoxide radical detection by NBT staining and phe confocal microscopy detection. *Spartina alterniflora* leaves were grown on 0.5 MS medium supplemented with 400 µm phe for 2 days. NBT detection highlighted the superoxide radical production, observed in salt glands on the adaxial leaf surface **(A)** and in leaf semi-thin section (**B**; 1: cap cell, 2: basal cell). Free phe is detected by confocal fluorescence microscopy (CFM; **C**), revealed by intracellular purple spots in these structures (the red color corresponds to chlorophyll fluorescence). Scale bars = 50 µm.

Similar experiments were performed in species from *Spartina*, described to be highly tolerant to PAHs (Alvarez et al., 2018; Cavé-Radet et al., 2019). Here, we focused on the two hexaploid parents *S. maritima* and *S. alterniflora*, and the allododecaploid *S. anglica* which resulted from genome doubling of their interspecific hybrid. Leaves of these three *Spartina* species were grown under high phe concentration (400 µm), followed by Nitroblue Tetrazolium (NBT) staining. UV light microscope observations revealed that superoxide radical production was mostly restricted to salt glands, mainly distributed along the adaxial leaf surface (**Figure 2A**). Basically, salt glands (also called hydathodes) are specialized structures involved in salt accumulation and excretion in *Spartina* and other Poaceae species (Skelding and Winterbotham, 1939; Barhoumi et al., 2008). In order to understand the structure of these particular cells and to verify if there is any co-localization of phe with this excess production of superoxide radicals, we investigated semi-thin leaf sections (**Figure 2B**). As first described by Skelding and Winterbotham (1939), we observed that salt glands include two distinct cells, a small cap cell (**Figure 2B-1**) inserted into the epidermis and a much larger basal cell (**Figure 2B-2**). Confocal microscopy analysis of thin sections of phe-treated leaf sections did not detect any phe signal in the cap cells, in contrast to the basal cells where strong intracellular fluorescence patches associated to phe sequestration were found (**Figure 2C**).

According to our results, we assume that trichomes, pavement cells and the basal cells of salt glands are potentially specialized cells involved in the accumulation and/or dissipation of organic xenobiotics in higher plants. Such observations are corroborated by the inorganic detoxification potentials previously reported in trichomes (Hall, 2002; Wienkoop et al., 2004) and salt glands (Thomson, 1975; Kraus et al., 1986; Burke et al., 2000). In response to organic xenobiotics, our results are consistent regarding the recent reports by Alves et al. (2017) about PAH cellular localization in *Medicago sativa*, where authors revealed phe and other PAHs accumulated in glandular secreting trichomes. However, to our knowledge the putative impact of pavement cells in xenobiotic detoxification is still missing in the literature.

At the molecular level, several studies provided evidences that trichomes although being non-secreting unicellular structures in *Arabidopsis*, are central accumulating and/or detoxifying structures. In fact, Gutierrez-Alcala et al. (2000) reported up to 300-fold more glutathione (GSH) content in trichomes as compared to other epidermal cells. As GSH are expected to be involved in ROS scavenging and xenobiotic detoxification (Das and Roychoudhury, 2014), such feature supports the high conjugation potential of trichomes. Moreover, the enrichment in peroxidases and CYPs involved in xenobiotic hydroxylation (Aziz et al., 2005), and the identification of detoxifying molecules under sulfur-induced stress (Wienkoop et al., 2004) in *Arabidopsis* trichomes are consistent with our observations. Complementary results were also reported in trichomes of tobacco, where expression profiling identified genes involved in responses to abiotic and biotic stresses, specifically expressed under cadmium exposure (Harada et al., 2010).

### Comparative Salt Gland Features and PAH Tolerance Following Allopolyploidization in *Spartina*

In a previous study, we reported enhanced tolerance to PAHs in *S. anglica* compared to the parental species *S. alterniflora* and *S. maritima* (Cavé-Radet et al., 2019). In this paper, we used comparative analyses of phe and ROS cellular compartmentalization, and photosynthetic indices, to highlight enhanced tolerance to phe in *S. anglica* following allopolyploidization. Other supplementary results in accordance with these reports are provided in this manuscript as supplementary data. On leaves grown one month under 100 µm phe (as described in the materials & methods section), we observed that *S. maritima* and *S. alterniflora* turned senescent indicating a high degradation and cell death phenotype, unlike *S. anglica* (**Supplementary Figure S1**). Even cultivated under 400 µm phe for 10 days, we did not measure by spectrometry a significant reduction in photosynthetic pigment contents (chlorophylls and carotenoids) in *S. anglica* in contrast to the parental species (**Supplementary Table S1**).

**Figure 3:**
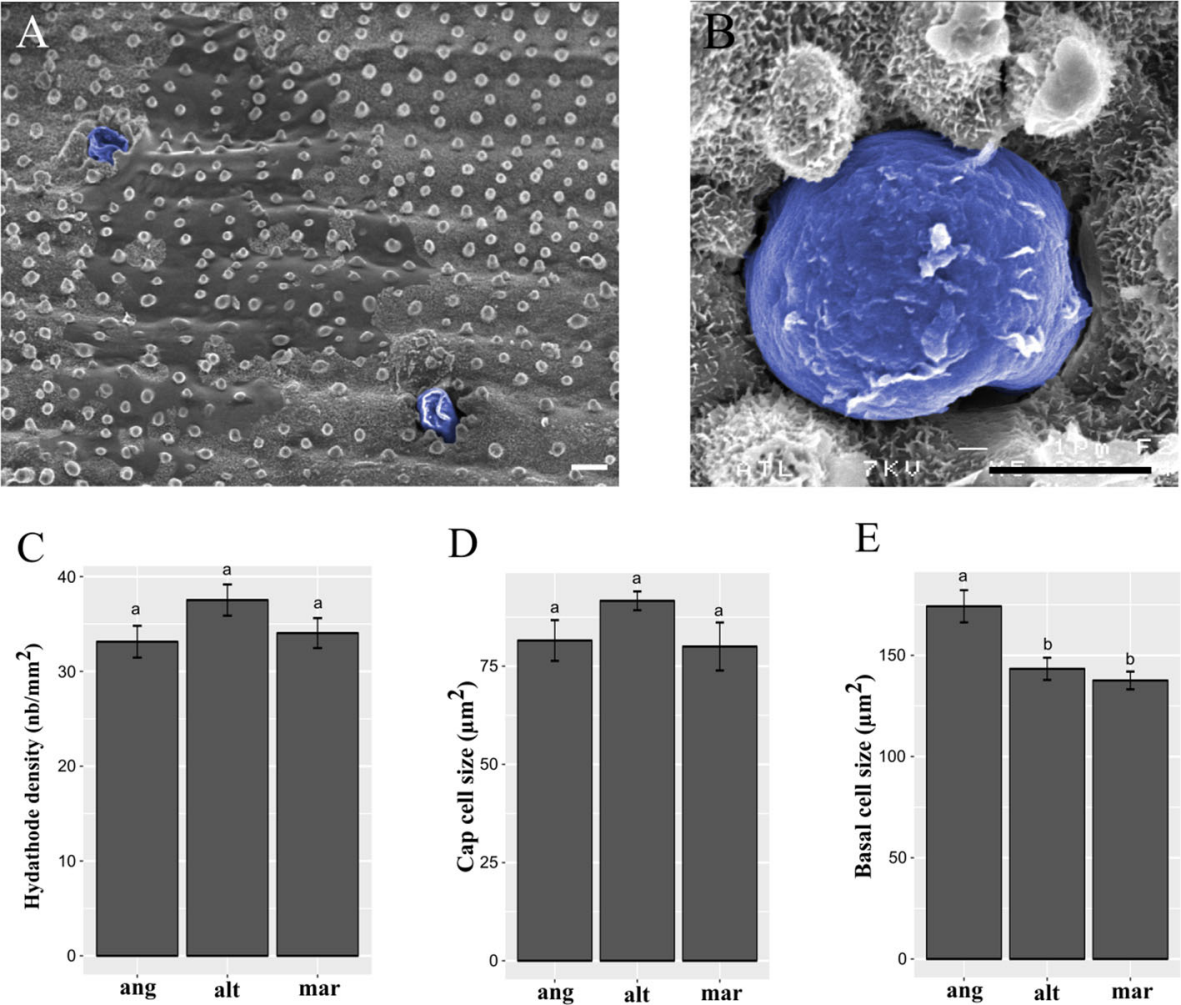
Spartina salt gland structure and observation. Salt glands were observed in ridges of the adaxial leaf surface by scanning electron microcopy, and colored in blue after taking photographs **(A**, **B)**. Scale bars = 5 µm. We measured salt gland densities (**C**; in gland.mm^-2^), cap cell size (**D**; in µm^2^), and basal cell size (**E**; in µm^2^) in the allododecaploid *Spartina anglica* (ang) and its related parents *S. alterniflora* (alt) and *S. maritima* (mar). Values annotated with different letters between species are significantly different according to Kruskal-Wallis’s multiple comparison test (with Bonferroni correction, p.value < 0.05).

In order to find out if there is any relationship between salt gland features and phe tolerance abilities, we compared salt gland densities and cell sizes between species. Using both UV light and scanning electron microscopy, we performed the measures and provided high quality images of salt glands along the adaxial leaf surface (**Figure 3A**), and cap cells of salt glands in the leaf ridges (**Figure 3B**). No significant difference in salt gland density was observed between species, with average densities of 37.5 ± 1.6 gland.mm^-2^ for *S. alterniflora*, 34.0 ± 1.6 for *S. maritima* and 33.1 ± 1.7 for *S. anglica* (**Figure 3C**). Similarly, no significant difference in the size of the cap cell of the salt gland was observed (average size of cap salt gland cells of 91.6 ± 3.4 µm^2^ in *S. alterniflora,* 80.0 ± 6.0 µm^2^ in *S. maritima* and 81.5 ± 5.1 µm^2^ in *S. anglica*; **Figure 3D**). By contrast, significant differences were found for basal cells, with larger basal cells found in *S. anglica* (174.2 ± 8.0 µm^2^) as compared to the parental species (143.3 ± 5.5 in *S. alterniflora* and 137.6 ± 4.4 in *S. maritima;* **Figure 3E**).

Our results provide a positive correlation between the size of basal salt gland cells and phe tolerance abilities previously reported following allopolyploidization in *Spartina* (Cavé-Radet et al., 2019). One can assume that genome doubling in *S. anglica* may increase cell size, as we observed on basal salt gland cells compared to the parental species. Surprisingly, our investigations did not show any differences between cap cell sizes from hexaploid and dodecaploid *Spartina* species. Hence, we assume that larger basal cells in *S. anglica* may be related with enhanced tolerance to phe (among allopolyploidy evolutionary novelties), for example through increased metabolism and storage capacities while no differences in salt gland densities were reported between species. In combination with previous observations, these data are consistent with the function of salt glands as central PAH detoxifying structures (*e.g.* storage/metabolization). Therefore, complementary analyses are needed to validate the role of basal salt gland cells in response to organic xenobiotic-induced stress. In *Spartina*, cell specific transcriptome profiling would be of high interest to conduct gene set enrichment analysis of basal salt gland cells between species. This would provide molecular data to characterize gene expression profiles in larger basal salt gland cells of *S. anglica* compared to the parental species, especially concerning genes involved in the xenome.

### *Arabidopsis* Trichomeless Mutants Exhibit Sensitive Phenotypes Under Phe-Induced Stress

**Figure 4:**
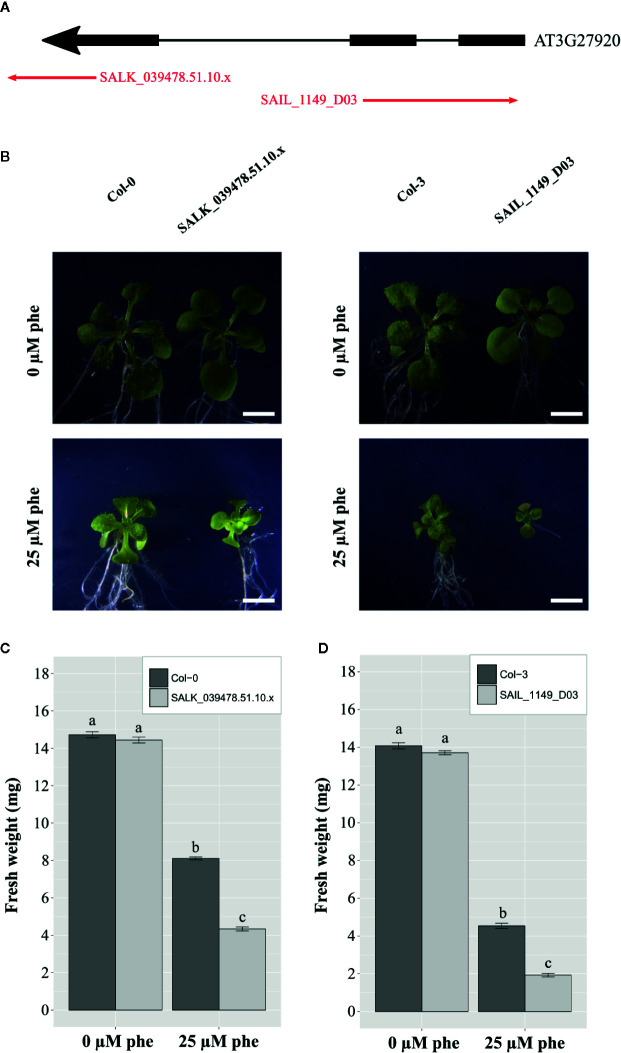
Functional validation of trichomes in PAH tolerance mechanisms in *A. thaliana*. T-DNA insertion mutant lines (SALK_039478.51.10.x and SAIL_1149_D03) inserted in exons of GLABRA 1 gene (AT3G27920: MYB protein domain involved in trichome development) were selected **(A)**. Phenotypical changes of the mutants compared to the background ecotypes under control (0 µm phe) and phe-induced stress (25 µm phe) were observed, and 21-day-old representative plantlets were shown **(B)**. Fresh weight of the mutants compared to the background ecotype under control (0 µm phe) and phe-induced stress (25 µm phe) in 21-day-old plantlets are presented for SALK_039478.51.10.x **(C)**, and SAIL_1149_D03 **(D)**. Means are calculated from three biological replicates; bars correspond to the standard errors. Values annotated with different letters are significantly different according to Kruskal-Wallis multiple comparison’s test (with Bonferroni correction, p.value < 0.05). Scale bars = 2 mm.

To test the possible impact of trichomes in organic xenobiotic detoxification, we selected two independent homozygous *Arabidopsis* glabrous mutant lines SALK_039478.51.10.x and SAIL_1149_D03, which presented T-DNA insertions in the GLABRA1 gene (AT3G27920: MYB protein domain involved in trichome development, **Figure 4A**). Glabrous mutant plants are characterized by the absence of trichomes. Plantlets were cultivated under control and phe-induced stress (0 and 25 µm) and compared to their background ecotypes. After 21 days, plantlets were sampled, weighed, and photographed. Representative plantlets (**Figure 4B**) and fresh weights plots are presented (**Figures 4C, D**).

Concerning SALK_039478.51.10.x mutants, plantlet weight was 13.7 ± 0.1 mg under control conditions, and was dramatically reduced (1.9 ± 0.1 mg) under phe-induced stress, while plantlet weight of the corresponding background ecotype Col-0 was 14.1 ± 0.2 mg under control conditions and 4.5 ± 0.1 mg under 25 µm of phe (**Figure 4C**). The same pattern was observed in plantlet weight of SAIL_1149_D03 (14.4 ± 0.2 mg under control conditions and 4.3 ± 0.1 mg under phe-induced stress), and in the corresponding background ecotype Col-3 (14.7 ± 0.2 mg under control conditions against 8.1 ± 0.1 mg under 25 µm of phe; **Figure 4D**). However, the biomass of both mutants was significantly reduced under phe-induced stress as compared to background ecotypes (**Figures 4C, D**).

In *Arabidopsis*, a moderate phe-induced stress (25 µm) induces a significant reduction in plant development. Here, the development of glabrous mutants was further reduced as compared to their background ecotypes under phe-induced stress, which further confirms that trichomes are most likely specialized cells involved in organic xenobiotic detoxification as hypothesized above.

### The Putative Advantages of Endopolyploidy in Specialized Cells Involved in Xenobiotic Detoxification

Altogether, these data suggest that in higher plants, cells of the whole organism do not respond equally to phe-induced stress, and that some specialized cells such as trichomes, pavement cells, and basal salt gland cells appear as key components in response to organic xenobiotic-induced stress. Plant specialized cells in detoxification should have increased transcriptomic, metabolic, and morphological abilities to detoxify organic xenobiotics. Interestingly, both cell lineages, trichomes and pavement cells, are known to undergo several rounds of genome doubling without the initiation of mitosis (Melaragno et al., 1993; Schnittger and Hülskamp, 2002; Katagiri et al., 2016). This phenomenon is known as somatic polyploidy, endoreduplication or endopolyploidy (Bateman et al., 2018), and may be of interest for such features. In fact, Barkla et al. (2018) pointed out that “highly metabolically active cells appear to have the highest levels of endopolyploidy, as it has been demonstrated in the endosperm and suspensor cells of the seeds of *Arum maculatum* and *Phaseolus coccineus* (24,567 and 8,192C, respectively; Leitch and Dodsworth, 2017)”. Despite a majority of tissues within *Arabidopsis* endoreduplicate at a basal level (Buchanan et al., 2000), it is important to keep in mind that endopolyploidy-associated growth is sometimes confined to specialized cell types or tissues that perform specific biological functions (*e.g.* the endosperm in sorghum, see Kladnik et al., 2006; or the pericarp in tomato, see Renaudin et al., 2017; for a review see Barkla et al., 2018). This mostly concerns localized endopolyploids, for which endopolyploidy is punctual in opposition with systemic endopolyploid species like *Arabidopsis*. In the beginning of their formation, *Arabidopsis* trichomes cease mitotic divisions and start a multitude of endoreduplication cycles. This multiplies their DNA content several times over, increases their size, and modifies their growth direction (Melaragno et al., 1993; Hülskamp et al., 1994; Kasili et al., 2011). The macroscopic plant trichomes in *Arabidopsis* are unicellular hairs displaying a high number of genomic copies ranging from 8 to 64C, and pavement cells may reach 16C (Melaragno et al., 1993; Schnittger and Hülskamp, 2002; Hülskamp, 2004).

In contrast to polyploidy, where the duplicated DNA is inherited through the germline and perpetuated over subsequent generations in all cells (Ainouche and Jenczewski, 2010), endopolyploidy arises from variations in the cell cycle where the genome is replicated without cell division, resulting in cells with a single polyploid nucleus and increased DNA content. The increase in DNA content often correlates with increased cell size (Robinson et al., 2018). According to Kondorosi et al. (2000), the increase in cell sizes is widespread in plants and represents a characteristic of endopolyploid cells; the higher the ploidy level, the larger the polyploid cell.

Interestingly, polyploid plants (inherited ploidy) can undergo endoreduplication (somatic polyploidy), as reported for example in trichomes from tetraploid *Arabidopsis* (del Pozo and Ramirez-Parra, 2015). In *Spartina*, Levering and Thomson (1971) described the ultra-structural salt gland cells of *Spartina*, and pointed out that basal salt gland cells contain a very large nucleus (also observed in salt glands from other Poaceae by Somaru et al., 2002). Hence, we hypothesized that basal salt gland cells from polyploid *Spartina* species still likely undergo endoreduplication, in association with the enlargement of the nucleus and cell size. Beside we reported basal salt gland cells enlargement in the dodecaploid compared to the hexaploid *Spartina* species, we assumed that both processes may overlap (inherited polyploidy and endoreduplication). This would imply higher ploidy levels in basal salt gland cells compared to other cells for which hexaploid cytotypes in *S. alterniflora* and *S. maritima* and dodecaploid cytotypes in *S. anglica* are expected. In this perspective, endoreduplication in basal salt gland cells could be related with enhanced xenobiotic storage/metabolization potentials (in addition to evolutionary benefits potentially raised by polyploidy), as proposed concerning *Arabidopsis* trichomes or pavement cells. To date, the relationship between inherited polyploidy and endopolyploidy is still unclear, but Pacey et al. (2020) provided recent evidences that polyploidy reduces endopolyploidy (by comparing *Arabidopsis* diploid vs. colchicine-treated tetraploid accessions). However, such trade-off was not currently reported between all *Arabidopsis* genotypes considered by Scholes (2020), depicting that endopolyploidy affected by inherited polyploidy is genotype dependent, and may also increase specifically in tetraploid plants in response to stress damage. Thus, further studies in distant plant lineages such as *Spartina* would be of interest to decipher how inherited polyploidy combined with endopolyploidy may be beneficial in response to xenobiotics, with a special focus on salt glands. Hence, measurements in ploidy levels of basal salt gland cells between *Spartina* species are needed to confirm such hypothesis.

Here, we reviewed the putative advantages given by endopolyploidy in specialized cells (largely reported in *Arabidopsis* trichomes and pavement cells, and hypothesized in *Spartina* basal salt gland cells) involved in xenobiotic detoxification. We think that endoreduplication represents an interesting scope for future analyses studying the role of specialized cells in xenobiotic detoxification. Notably, it has been suggested that the impact of endopolyploidy is most likely related with an increase in gene expression to sustain high protein production and metabolic activity necessary for development, cell enlargement, or stress response (Scholes and Paige, 2015; Schoenfelder and Fox, 2015). In *Arabidopsis* trichomes, endoreduplication may explain the enrichment in detoxifying molecules previously described in these structures (Wienkoop et al., 2004; Aziz et al., 2005).

## Conclusion

In the present work, we provide observations assuming that specialized cells are key components involved in organic xenobiotic sequestration, metabolization and/or dissipation from PAHs. This includes trichomes and pavement cells in *Arabidopsis* and the basal salt gland cells in *Spartina*. Our observations were supported by (1) a positive correlation between the size of basal salt gland cells and phe tolerance abilities between *Spartina* species, and (2) a reverse genetic study of trichomeless *Arabidopsis* mutants depicting sensitive phenotypes to phe. Hence, we shed new insights into the understanding of xenobiotic detoxification in higher plants, and more investigations addressing cell specific responses through time and plant species are needed to dissect the role of such specialized cells in this process, especially concerning *Arabidopsis* pavement cells.

In complement, we discussed the putative advantages given by endoreduplication, increasing specific metabolic pathways or developmental process in such specialized cells. In endopolyploid cells, a positive relationship between cell size, nucleus size, and ploidy level is largely reported. Even if Tsukaya (2019) mentioned recently that cell size could be controlled genetically and not solely by endopolyploidy in *Arabidopsis*, several investigations stated clearly that trichomes and pavement cells undergo endoreduplication. In *Spartina*, we hypothesized that the basal salt gland cells may undergo endoreduplication too (higher ploidy levels than respectively expected in hexaploid or dodecaploid species), improving their xenobiotic detoxification abilities. However, the measurements of endopolyploidy remain to be experimentally demonstrated in further research, as Scholes and Paige (2014) reported that the levels of ploidy may increase through time in some ecotypes of *Arabidopsis*, and particularly under stress/damage. In *Spartina*, such analyses could provide supplemental data to decipher the role of inherited polyploidy in combination with endopolyploidy in the context of recent natural allopolyploidization event.

In animal systems, detoxification of xenobiotics occurred mainly in endopolyploid liver cells. Interestingly, the triggering of xenobiotic detoxifying systems with increased level of endopolyploidy was observed in mammal liver (Sanz et al., 1997). This intriguing similarity might be explained by the fact that endopolyploidy increase transcription. This hypothesis has been already clearly demonstrated by Bourdon et al. (2012), as they provide direct evidence that endopolyploidy play a role in increased transcription in tomato pericarp cells. In response to xenobiotics, we assumed that increased xenome transcription can be decisive in regard to the putative role of specialized cells in xenobiotic detoxification strategies.

## Materials and Methods

### Plant Material and Experimental Design

To detect if specialized are involved in organic xenobiotic detoxification in higher plants, we focused our analyses in two contrasting plant lineages, using the diploid plant model *Arabidopsis thaliana* and species from the polyploid *Sporobolus* genus subsect. *Spartina* (*Spartina* Schreb. Clade in Poaceae). In *Spartina*, we selected the parental hexaploid species *S. alterniflora* Loisel (2n = 6x = 62) and *S. maritima* Curtis (2n = 6x = 60), and their allopolyploid derivative *S. anglica* C.E. Hubbard (2n = 12x = 120–124) which resulted from genome doubling of their interspecific hybrid. Phe is a common PAH we used throughout the study. *Arabidopsis* represents a sensitive species to xenobiotics, with a sub-lethal phe concentration of 200 µm (Dumas et al., 2016). In comparison, *Spartina* species are much more tolerant (*S. anglica* can tolerate even up to 800 μm phe). The three species were selected because enhanced tolerance abilities to phe were previously reported following allopolyploidization in *S. anglica* (Cavé-Radet et al., 2019).

Seeds of *Arabidopsis thaliana* (Col-0) were germinated in Petri dishes on a solid growth medium (half-strength Murashige and Skoog: 0.5 MS; 0.8% agar; pH = 5.6). To ensure that phe is translocated upwards from roots to leaves *via* the xylem stream, plantlets were grown vertically for 6 days, and then transferred on an identical medium supplemented with 0 and 25 µm phe solubilized in absolute ethanol, with only roots being in contact with the medium. A sterile transparent plastic film was applied between plantlets and the medium to avoid any contact of the rosette-leaves with phe. In total, *Arabidopsis* plantlets were cultivated *in vitro* for 21 days before experiments, in phytotronic chambers under fluorescent light/dark regime of 16/8 h, an average ambient temperature of 22/20°C, a light intensity of 8500 Lux and a relative humidity of 60%.

In complement, fragments of *Spartina* leaves were cultivated using a method previously described in Cavé-Radet et al. (2019). Here, we cultivated leaves from the three species for 2 days in petri dishes on the solid growth medium as previously described for *Arabidopsis* (0 and 400 µm phe, three biological and technical replicates).

### Cellular Localizations of phe and ROS in *Arabidopsis* and *Spartina*

In *Arabidopsis*, at least 6 plantlets per treatment were filtered under vacuum with 100 µm SOSG reagent in 50 µm phosphate potassium buffer (pH = 7.5). Observations were performed using a spectrofluorometer to reveal singlet oxygen radicals in leaf tissues by green fluorescence, and coupled with epifluorescence microscopy detection (EFMD) to reveal phe.

In *Spartina*, at least 6 leaves per treatment and species were filtered under vacuum with 35 mg.ml^-1^ NBT in phosphate potassium buffer (10 mm, pH = 7.5), followed by three hours of reaction. Then, leaves were immersed for 15 min in acetic-glycerol-ethanol solution (v. 1:1:3) at 100°C, before being bleached in 80% acetone until complete pigment discoloration. Observations were performed using a UV light microscopy to reveal superoxide radicals by blue fluorescent patches in leaf tissues (along the adaxial surface or in semi-thin sections), and coupled with confocal microscopy (CFM) to reveal phe.

Here, we used both singlet oxygen and superoxide radicals as oxidative stress markers (respectively in *Arabidopsis* and *Spartina*). As well, phe is detected either using EFMD or CFM by its blue fluorescence emitting from 420–480 nm with a weak but specific signal around 430 nm on 405 nm excitation. As a free molecule, phe is easily visualized under fluorescence, and we took advantage of this to screen specific phe accumulation in a myriad of cells in the two contrasting plant lineages.

### Salt Gland Densities and Cell Sizes in *Spartina* Species

To provide additional data supporting that salt glands in *Spartina* may represent key structures involved in organic xenobiotic detoxification, we aimed to measure salt gland densities and cell sizes between the three species from divergent tolerance abilities to phe (Cavé-Radet et al., 2019). In *Spartina* leaves cultivated under 400 µm phe, NBT staining allowed us to identify highly localized coloration showing ROS production, specifically in salt glands along the adaxial leaf surface. Thus, at least 6 replicates of phe-treated leaves filtered with NBT were photographed, and blue-stained salt glands were counted inside areas from 0.8 to 2.6 mm^2^ to calculate densities. Salt glands were similarly observed in semi-thin sections on the same samples by UV light microscopy, and basal cell sizes were measured. In addition, cap salt gland cells were observed by scanning electron microscopy (SEM, platform CMEBA UR1). Here, at least 6 replicates of fresh leaves were dehydrated in successive diluted ethanol solutions (70, 80, 90, and 100%) for 10 min. Because salt glands are mainly encountered in the leaf ridges along the adaxial surface, leaves were fractured in liquid nitrogen and fixed prior to SEM observation. Then, cap cells were photographed and measured. Image processing was performed using ImageJ software (v 1.51j8). Statistical analyses were performed between species by pairwise comparisons using Kruskal-Wallis non-parametric test in R 3.5 (R Core Team, 2015).

### Functional Validation of Trichomes in Organic Xenobiotic Tolerance Mechanisms

To validate the impact of trichomes in organic xenobiotic tolerance mechanisms, a functional validation was performed using T-DNA mutant lines affecting GLABRA1 expression (AT3G27920) which disrupt trichome development. Homozygous glabrous mutant (trichomeless) lines SALK_039478.51.10.x (Col-0 background ecotype) and SAIL_1149_D03 (Col-3 background ecotype) were selected (SIGnAL database, http://signal.salk.edu/). Mutants were grown *in vitro* in Petri dishes (0 and 25 µm phe), stocked for 48h at 4°C before being transferred into phytotronic chambers (same culture protocol and conditions as described for *Arabidopsis* plantlets in section 4.1). The experiment was performed in biological triplicates. After 21 days, 10 plantlets per treatment were sampled and weighed. Pairwise comparisons were performed between treatments, and mutants were compared to their background ecotypes using Kruskal-Wallis non-parametric tests in R 3.5 (R Core Team, 2015).

## Data Availability Statement

The datasets generated for this study are available on request to the corresponding authors.

## Author Contributions

AE conceived the experiments. AE, AC-R, and FG performed the experiments. AE, AC-R, and MR analyzed the data. AE, AC-R, and MR wrote the manuscript. All authors contributed to the article and approved the submitted version.

## Funding

This work was supported by the “Ministère de l’Enseignement Supérieur et de la Recherche”, the CNRS, and the “Observatoire des Sciences et de l’Univers de Rennes” (OSUR) funds.

## Conflict of Interest

The authors declare that the research was conducted in the absence of any commercial or financial relationships that could be construed as a potential conflict of interest.

## References

[B1] Ainouche M. L. Jenczewski E. (2010). Focus on polyploidy. New Phytol. 186 (1), 1–4. 10.1111/j.1469-8137.2010.03215.x 20409175

[B2] Alkio M. Tabuchi T. M. Wang X. Colon-Carmona A. (2005). Stress responses to polycyclic aromatic hydrocarbons in *Arabidopsis* include growth inhibition and hypersensitive response-like symptoms. J. Exp. Bot. 56, 2983–2994. 10.1093/jxb/eri295 16207747

[B3] Alvarez M. Ferreira de Carvalho J. Salmon A. Ainouche M. L. Cavé-Radet A. El Amrani A. (2018). Transcriptome response of the foundation plant Spartina alterniflora to the Deepwater Horizon oil spill. Mol. Ecol. 27, 2986–3000 10.1111/mec.14736 29862597

[B4] Alves W. S. Manoel E. A. Santos N. S. Nunes R. O. Domiciano G. C. Soares M. R. (2017). Detection of polycyclic aromatic hydrocarbons (PAHs) in *Medicago sativa* L. by fluorescence microscopy. Micron 95, 23–30. 10.1016/j.micron.2017.01.004 28178583

[B5] Aziz N. Paiva N. L. May G. D. Dixon R. A. (2005). Transcriptome analysis of alfalfa glandular trichomes. Planta 221, 28–38. 10.1007/s00425-004-1424-1 15578217

[B6] Barhoumi Z. Djebali W. Abdelly C. Chaibi W. Smaoui A. (2008). Ultrastructure of *Aeluropus littoralis* leaf salt glands under NaCl stress. Protoplasma 233, 195–202. 10.1007/s00709-008-0003-x 18563515

[B7] Barkla B. J. Rhodes T. Tran K. T. Wijesinghege C. Larkin J. C. Dassanayake M. (2018). Making epidermal bladder cells bigger: developmental- and salinity-induced endopolyploidy in a model halophyte. Plant Physiol. 177 (2), 615–632. 10.1104/pp.18.00033 29724770 PMC6001328

[B8] Bateman R. M. Guy J. J. Rudall P. J. Leitch I. J. Pellicer J. Leitch A. R. (2018). Evolutionary and functional potential of ploidy increase within individual plants: somatic ploidy mapping of the complex labellum of sexually deceptive bee orchids. Ann. Bot. 122 (1), 133–150. 10.1093/aob/mcy048 29672665 PMC6025197

[B9] Bourdon M. Pirrello J. Cheniclet C. Coriton O. Bourge M. Brown S. (2012). Evidence for karyoplasmic homeostasis during endoreduplication and a ploidy-dependent increase in gene transcription during tomato fruit growth. Development 139, 3817–3826. 10.1242/dev.084053 22991446

[B10] Buchanan B. B. Gruissem W. Jones R. L. (Eds.) (2015). Biochemistry & molecular biology of plants. Second edition. Chichester, West Sussex. Hoboken, NJ: John Wiley & Sons Inc.

[B11] Burke D. J. Weis J. S. Weis P. (2000). Release of metals by the leaves of the salt marsh grasses *Spartina alterniflora* and *Phragmites australis* . Estuar. Coast. Shelf Sci. 51, 153–159. 10.1006/ecss.2000.0673

[B12] Cavé-Radet A. Salmon A. Lima O. Ainouche M. El Amrani A. (2019). Increased tolerance to organic xenobiotics following recent allopolyploidy in *Spartina* (Poaceae). Plant Sci. 280, 143–154. 10.1016/j.plantsci.2018.11.005 30823992

[B13] Collins C. D. Martin I. Doucette W. (2011). “Plant Uptake of Xenobiotics”, in Organic Xenobiotics and Plants. Eds. Schröder P. Collins C. D. (Dordrecht: Springer Netherlands), 3–16.

[B14] Das K. Roychoudhury A. (2014). Reactive oxygen species (ROS) and response of antioxidants as ROS-scavengers during environmental stress in plants. Front. Environ. Sci. 2, 53. 10.3389/fenvs.2014.00053

[B15] del Pozo J. C. Ramirez-Parra E. (2015). Whole genome duplications in plants: An overview from *Arabidopsis* . J. Exp. Bot. 66, 6991– 7003. 10.1093/jxb/erv432 26417017

[B16] Dumas A. S. Taconnat L. Barbas E. Rigaill G. Catrice O. Bernard D. (2016). Unraveling the early molecular and physiological mechanisms involved in response to phenanthrene exposure. BMC Genomics 17 (1), 818. 10.1186/s12864-016-3133-0 27769163 PMC5073745

[B17] Edwards R. Brazier-Hicks M. Dixon D. P. Cummins I. (2005). “Chemical manipulation of antioxidant defenses in plants”, in Advances in Botanical Research (United States: Elsevier), 42, 1–32. 10.1016/s0065-2296(05)42001-7

[B18] Edwards R. Dixon D. P. Cummins I. Brazier-Hicks M. Skipsey M. (2011). “New perspectives on the metabolism and detoxification of synthetic compounds in plants” in Organic Xenobiotics and Plants. Eds. Schröder P. Collins C. D. (Dordrecht: Springer Netherlands), 125–148.

[B19] El Amrani A. Dumas A. S. Wick L. Y. Yergeau E. Berthomé R. (2015). “Omics” insights into PAH degradation toward improved green remediation biotechnologies. Environ. Sci. Technol. 49, 11281–11291. 10.1021/acs.est.5b01740 26352597

[B20] Gutierrez-Alcala G. Gotor C. Meyer A. J. Fricker M. Vega J. M. Romero L. C. (2000). Glutathione biosynthesis in *Arabidopsis* trichome cells. PNAS 97, 11108–11113. 10.1073/pnas.190334497 10995473 PMC27156

[B21] Hülskamp M. Misera S. Jürgens G. (1994). Genetic dissection of trichome cell development in *Arabidopsis* . Cell 76, 555–566. 10.1016/0092-8674(94)90118-x 8313475

[B22] Hülskamp M. (2004). Plant trichomes: a model for cell differentiation. Nat. Rev. Mol. Cell. Biol. 5, 471–480. 10.1038/nrm1404 15173826

[B23] Hall J. L. (2002). Cellular mechanisms for heavy metal detoxification and tolerance. J. Exp. Bot. 53, 1–11. 10.1093/jxb/53.366.1 11741035

[B24] Harada E. Kim J. A. Meyer A. J. Hell R. Clemens S. Choi Y. E. (2010). Expression profiling of tobacco leaf trichomes identifies genes for biotic and abiotic stresses. Plant Cell Physiol. 51, 1627–1637. 10.1093/pcp/pcq118 20693332

[B25] Kasili R. Huang C. C. Walker J. D. Simmons L. A. Zhou J. Faulk C. (2011). Branchless trichomes links cell shape and cell cycle control in *Arabidopsis* trichomes. Development 138, 2379–2388. 10.1242/dev.058982 21558384 PMC6512272

[B26] Katagiri Y. Hasegawa J. Fujikura U. Hoshino R. Matsunaga S. Tsukaya H. (2016). The coordination of ploidy and cell size differs between cell layers in leaves. Development 143, 1120–1125. 10.1242/dev.130021 26903507 PMC4852493

[B27] Kladnik A. Chourey P. S. Pring D. R. Dermastia M. (2006). Development of the endosperm of *Sorghum bicolor* during the endoreduplication-associated growth phase. J. Cereal Sci. 43, 209–215. 10.1016/j.jcs.2005.09.004

[B28] Kolb D. Müller M. Zellnig G. Zechmann B. (2010). Cadmium induced changes in subcellular glutathione contents within glandular trichomes of *Cucurbita pepo* L. Protoplasma 243, 87–94. 10.1007/s00709-009-0043-x 19424775 PMC2892058

[B29] Kondorosi E. Roudier F. Gendreau E. (2000). Plant cell-size control: growing by ploidy? Curr. Opin. Plant Biol. 3, 488– 492. 10.1016/S1369-5266(00)00118-7 11074380

[B30] Kraus M. L. Weis P. Crow J. H. (1986). The excretion of heavy metals by the salt marsh cord grass, *Spartina alterniflora*, and *Spartina*‘s role in mercury cycling. Mar. Environ. Res. 20, 307–316. 10.1016/0141-1136(86)90056-5

[B31] Kvesitadze E. Sadunishvili T. Kvesitadze G. (2009). Mechanisms of organic contaminants uptake and degradation in plants. World Acad. Sci. Eng. Technol. 55 (6), 458–468.

[B32] Leitch I. J. Dodsworth S. (2017). “Endopolyploidy in plants”, in eLS. Ed. John Wiley & Sons Ltd. (Chichester, UK: John Wiley & Sons, Ltd), 1–10. 10.1002/9780470015902.a0020097.pub2

[B33] Levering C. A. Thomson W. W. (1971). The Ultrastructure of the Salt gland of. Spartina Foliosa Planta 97, 183–196. 10.1007/BF00389200 24493239

[B34] Liu H. Weisman D. Ye Y. Cui B. Huang Y. Colón-Carmona A. (2009). An oxidative stress response to polycyclic aromatic hydrocarbon exposure is rapid and complex in *Arabidopsis thaliana* . Plant Sci. 176, 375–382. 10.1016/j.plantsci.2008.12.002

[B35] Melaragno J. E. Mehrotra B. Coleman A. W. (1993). Relationship between endopolyploidy and cell size in epidermal tissue of *Arabidopsis* . Plant Cell 5, 1661–1668. 10.1105/tpc.5.11.1661 12271050 PMC160394

[B36] Öztürk M. Ashraf M. Aksoy A. Ahmad M. S. A. Hakeem K. R. (2016). Plants, pollutants and remediation (New York, NY: Springer Berlin Heidelberg).

[B37] Pacey E. K. Maherali H. Husband B. C. (2020). The influence of experimentally induced polyploidy on the relationships between endopolyploidy and plant function in *Arabidopsis thaliana* . Ecol. Evol. 10, 198–216. 10.1002/ece3.5886 31988723 PMC6972801

[B38] R Core Team (2015). R: a language and environment for statistical computing (Vienne (Autriche): R foundation for statistical computing).

[B39] Renaudin J. P. Deluche C. Cheniclet C. Chevalier C. Frangne N. (2017). Cell layer-specific patterns of cell division and cell expansion during fruit set and fruit growth in tomato pericarp. J. Exp. Bot. 68, 1613–1623. 10.1093/jxb/erx058 28369617 PMC5444452

[B40] Robinson D. O. Coate J. E. Singh A. Hong L. Bush M. Doyle J. J. Roeder A. H. K. (2018). Ploidy and size at multiple scales in the *Arabidopsis* sepal. Plant Cell 30 (10), 2308–2329. 10.1105/tpc.18.00344 30143539 PMC6241276

[B41] Sanz A. Diez-Fernandez N. C. Alvarez A. Cascales M. (1997). Age-dependent modifications in rat hepatocyte antioxidant defense systems. J. Hepatol. 27, 525–534. 10.1016/S0168-8278(97)80358-3 9314131

[B42] Schnittger A. Hülskamp M. (2002). Trichome morphogenesis: a cell-cycle perspective. Philos. T. R. Soc B. 357, 823–826. 10.1098/rstb.2002.1087 PMC1692984 12079678

[B43] Schoenfelder K. P. Fox D. T. (2015). The expanding implications of polyploidy. J. Cell Biol. 209, 485–491. 10.1083/jcb.201502016 26008741 PMC4442802

[B44] Scholes D. R. Paige K. N. (2014). Plasticity in ploidy underlies plant fitness compensation to herbivore damage. Mol. Ecol. 23, 4862–4870. 10.1111/mec.12894 25145792

[B45] Scholes D. R. Paige K. N. (2015). Plasticity in ploidy: a generalized response to stress. Trends Plant Sci. 20, 165–175. 10.1016/j.tplants.2014.11.007 25534217

[B46] Scholes D. R. (2020). Ploidy in plant tolerance to apical meristem damage: a test of relative costs and benefits. Int. J. Plant Sci. 181, 509–517. 10.1086/707728

[B47] Shiri M. Rabhi M. Abdelly C. El Amrani A. (2015). The halophytic model plant *Thellungiella salsuginea* exhibited increased tolerance to phenanthrene-induced stress in comparison with the glycophitic one *Arabidopsis thaliana*: Application for phytoremediation. Ecol. Eng. 74, 125–134. 10.1016/j.ecoleng.2014.09.123

[B48] Skelding A. D. Winterbotham J. (1939). The structure and development of the hydathodes of *Spatina townsendii* groves. New Phytol. 38, 69–79. 10.1111/j.1469-8137.1939.tb07085.x

[B49] Somaru R. Naidoo Y. Naidoo G. (2002). Morphology and ultrastructure of the leaf salt glands of *Odysseapaucinervis* (Stapf) (Poaceae). Flora 197, 67–75. 10.1078/0367-2530-00016

[B50] Sun C. Dudley S. McGinnis M. Trumble J. Jay Gan J. (2019). Acetaminophen detoxification in cucumber plants via induction of glutathione S-transferases. Sci. Total Environ. 649, 431–439. 10.1016/j.scitotenv.2018.08.346 30176456

[B51] Taguchi G. Ubukata T. Nozue H. Kobayashi Y. Takahi M. Yamamoto H. (2010). Malonylation is a key reaction in the metabolism of xenobiotic phenolic glucosides in *Arabidopsis* and tobacco: Phenolic-xenobiotics metabolism in *Arabidopsis* . Plant J. 63, 1031–1041. 10.1111/j.1365-313X.2010.04298.x 20626660

[B52] Thomson W. W. (1975). “The Structure and function of salt glands” in Plants in Saline Environments. Eds. Poljakoff-Mayber A. Gale J. (Berlin, Heidelberg: Springer Berlin Heidelberg), 118–146.

[B53] Tsukaya H. (2019). Re-examination of the role of endoreduplication on cell-size control in leaves. J. Plant Res. 132, 571–580. 10.1007/s10265-019-01125-7 31321606 PMC6713683

[B54] Weisman D. Alkio M. Colón-Carmona A. (2010). Transcriptional responses to polycyclic aromatic hydrocarbon-induced stress in *Arabidopsis thaliana* reveal the involvement of hormone and defense signaling pathways. BMC Plant Biol. 10, 59. 10.1186/1471-2229-10-59 20377843 PMC2923533

[B55] Wienkoop S. Zoeller D. Ebert B. Simon-Rosin U. Fisahn J. Glinski M. (2004). Cell-specific protein profiling in *Arabidopsis thaliana* trichomes: identification of trichome-located proteins involved in sulfur metabolism and detoxification. Phytochemistry 65, 1641–1649. 10.1016/j.phytochem.2004.03.026 15276459

[B56] Zhan X. Zhang X. Yin X. Ma H. Liang J. Zhou L. (2012). H(+)/phenanthrene symporter and aquaglyceroporin are implicated in phenanthrene uptake by wheat (*Triticum aestivum* L.) roots. J. Environ. Qual. 41 (1), 188–196. 10.2134/jeq2011.0275 22218187

