# Supplementary material for: Do Specialized Cells Play a Major Role in Organic Xenobiotic Detoxification in Higher Plants?
Source: Front Plant Sci. 2020 Jul 9;11:1037. doi: 10.3389/fpls.2020.01037 (PMC7363956; doi:10.3389/fpls.2020.01037)
Supplement: Supplementary file 1 [file Image_1.pdf]

Supplementary figure

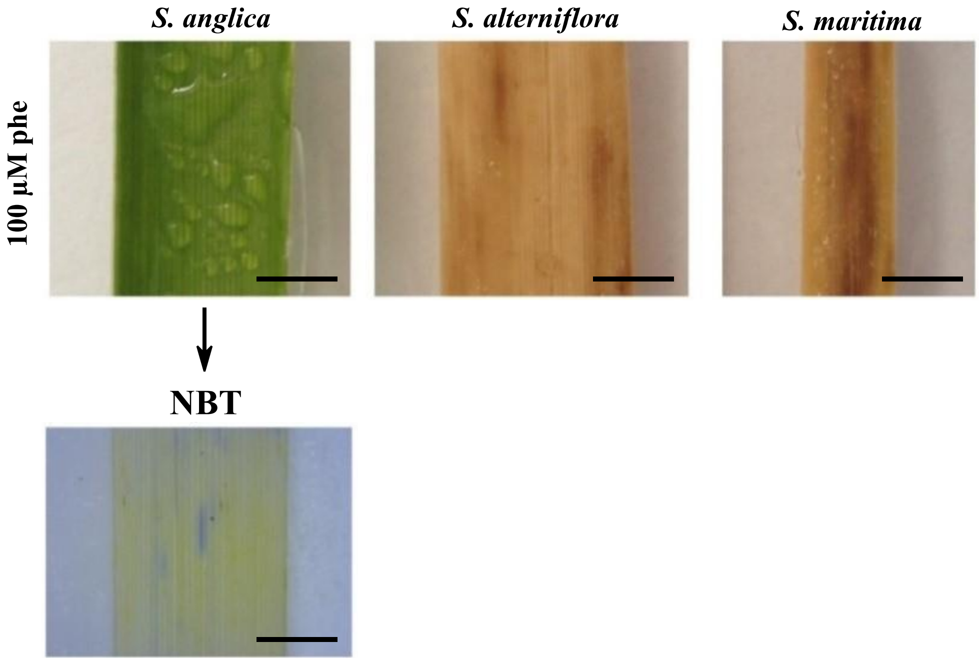

Supplementary Fig. S1. Phenotypical changes and superoxide radical production in *Spartina* leaves following long phe (100 µM) treatment (one month). *S. anglica* did not exhibit phenotypical stress markers, unlike the parental species *S. alterniflora* and *S. maritima* which lost all the photosynthetic pigment contents and turned senescent, indicating a high degradation and cell death phenotype. Scale bars = 5 mm.
