# Supplementary material for: Do Specialized Cells Play a Major Role in Organic Xenobiotic Detoxification in Higher Plants?
Source: Front Plant Sci. 2020 Jul 9;11:1037. doi: 10.3389/fpls.2020.01037 (PMC7363956; doi:10.3389/fpls.2020.01037)
Supplement: Supplementary file 2 [file Table_1.docx]

Supplementary Table

**Supplementary Tab. S1.** Photosynthetic pigment contents measured by spectrometry (expressed as % of the control) for each *Spartina* species and the MPV, treated for 10 days with 0, 100 and 400 µM phe. Each value (± SE) was evaluated from three biological replicates. For each species, values annotated with different letters in the same column are significantly different according to Duncan’s test (p.value < 0.05). MPV: MidParent Value (average value estimated by considering the two parental species combined), Chl (a+b), chlorophyll a and b, Car: carotenoids.

|  | ***S. alterniflora*** | | | ***S. maritima*** | | | **MPV** | | | ***S. anglica*** | | |
| --- | --- | --- | --- | --- | --- | --- | --- | --- | --- | --- | --- | --- |
| **phe (µM)** | 0 | 100 | 400 | 0 | 100 | 400 | 0 | 100 | 400 | 0 | 100 | 400 |
| **Chl (a+b)** | 100a | 93.6ab | 70.7b | 100a | 87.9ab | 54.4b | 100a | 90.8a | 64.2b | 100ab | 134.3a | 86.3b |
| **Car** | 100a | 85.5ab | 57.1b | 100a | 63.2a | 13.5b | 100a | 74.4ab | 39.6b | 100ab | 125a | 93.3b |
